# PurpleAir Sensor Deployment Trends and Uncertainties

**DOI:** 10.3390/s26061789

**Published:** 2026-03-12

**Authors:** Chloe S. Chung, Annette C. Rohr

**Affiliations:** Electric Power Research Institute, Palo Alto, CA 94304, USA

**Keywords:** low-cost air quality sensors, PurpleAir, PM2.5, sensor deployment

## Abstract

Low-cost air quality sensors, such as PurpleAir monitors, have rapidly expanded fine particulate matter (PM2.5) monitoring across the United States, providing dense, hyper-local measurements. While prior research has focused largely on sensor accuracy and calibration, less is known about where these sensors are deployed and whether they persist long enough to support multi-year analyses relevant to exposure assessment and policy. Using publicly available PurpleAir data, we characterized the geographic distribution, deployment longevity, and persistence of outdoor sensors across the United States from 2016 to 2025. We quantified deployment duration as the time between first and last publicly available observations and summarized patterns nationally, by U.S. Census region, and by state. Most publicly shared sensors remained deployed for more than three years, indicating substantial potential for multi-year applications, particularly in the western United States, where sensor density and longevity were highest. As an illustrative component, we present descriptive summaries of PM2.5 concentrations in four high-coverage states (California, Minnesota, Pennsylvania, and Texas) by deployment duration and urban–rural classification to demonstrate the types of analyses enabled by these networks. These results establish a national baseline of sensor availability and temporal continuity. By focusing on deployment patterns, this study provides foundational context for future exposure modeling, epidemiologic studies, and targeted expansion of community air quality monitoring networks.

## 1. Background

Low-cost air quality sensors, such as those produced by PurpleAir, have become widely deployed across the United States (U.S.) and internationally. These sensors provide real-time, hyper-local measurements of fine particulate matter (PM2.5), substantially expanding spatial coverage beyond regulatory monitoring networks. A growing body of validation studies has evaluated PurpleAir sensor performance relative to reference-grade monitors, demonstrating moderate-to-strong agreement after calibration while identifying systematic biases related to environmental conditions. Specifically, PurpleAir sensors rely on optical particle counters that are sensitive to relative humidity, temperature, and aerosol composition, often overestimating PM2.5 during wildfire smoke or high-humidity conditions; accordingly, laboratory-based, region-specific, and national corrections—including the U.S. EPA’s equation—have been developed [1,2,3] to address those issues. Beyond technical validation, these networks increasingly inform community engagement and situational awareness: a community-led effort in Waterbury, Connecticut, found strong agreement with reference monitors and supported local action [4], and the U.S. EPA integrates PurpleAir data into the national Fire and Smoke Map to inform real-time decisions during smoke events [5].

Despite these benefits, several constraints limit direct application to exposure assessment and policy. Deployments often lack standardized oversight and technical support; uncalibrated data can mislead users and may become nonlinear at very high concentrations (>300 µg/m^3^), complicating interpretation during severe events [6]. Many users also lack resources to deploy and interpret sensors appropriately, and common standards for reporting and metadata are still emerging [7,8]. Beyond performance, access is uneven: sensors cluster in higher-income, higher-education tracts, leaving disadvantaged communities with fewer monitors despite higher burdens [9,10,11]. A key open question is whether community deployments persist long enough to support long-term regulatory-relevant analyses that typically rely on multi-year (e.g., three-year) design values.

This study addresses this gap by quantifying the longevity of sensor operation, geographic coverage, and multi-year persistence of publicly shared PurpleAir sensors across the United States. In doing so, it complements prior work focused on sensor validation and calibration by providing a national-scale characterization of where low-cost sensors are deployed and how consistently they operate over time—information that is foundational for exposure modeling and policy-relevant applications.

Rather than focusing on concentration contrasts, the primary objective of this work is to establish this baseline understanding of sensor availability and temporal continuity, which is a necessary precursor for interpreting pollution patterns captured by these networks. As an illustrative component, we present PM_2.5_ summaries for selected states with high sensor density to demonstrate the types of analyses enabled by such deployment patterns. These summaries are intended to be descriptive and illustrative, rather than comprehensive exposure assessments, and are included to contextualize the deployment findings and inform future, dedicated air quality analyses using low-cost air quality sensor data.

## 2. Methods

### 2.1. Data Source and Fields

We obtained publicly available PurpleAir PM_2.5_ data via the PurpleAirAPI package in R. For each sensor, we extracted latitude, longitude, date_created, last_seen, location_type, humidity, temperature, pressure, and pm2.5_cf_1 (Table 1). We restricted the dataset to outdoor sensors (based on location_type) with valid geocoordinates.

To improve comparability across locations, we analyzed data from 2021 to 2024 (a period with broader network penetration).

### 2.2. Deployment Longevity Metric and Categorization

Our objective was to quantify operational deployment tenure—i.e., how long PurpleAir sensors remain deployed and discoverable on the public network—to assess suitability for multi-year use cases. We defined deployment duration for each outdoor device as Δt = last_seen−date_created from the PurpleAir API and classified sensors into <1 year, 1–2 years, 2–3 years, and >3 years categories. By design, this metric reflects the calendar data-presence window (first-to-last valid observation) and not verified continuous operation or sensor health; we did not infer hourly duty cycle, fill gaps, or relink moved/replaced devices. Results should therefore be interpreted as deployment tenure on the public network.

### 2.3. Regional Stratification and State-Level PM_2.5_ Characterization

We summarized deployment across U.S. Census regions (Northeast, South, Midwest, West) and by state. For illustrative PM_2.5_ characterization, we examined one state per region—the state with the largest number of outdoor sensors—to ensure sufficient sample size. This design provides high-coverage exemplars rather than region-wide representativeness; therefore, state-level PM_2.5_ summaries are presented descriptively, and we refrain from generalizing those distributions to entire regions. Our national conclusions about deployment longevity are based on the full dataset across all states.

To ensure data quality, we removed negative PM2.5 observations and values > 100 µg/m^3^; concentrations above ~100 µg/m^3^ in the U.S. are typically wildfire-smoke episodes rather than baseline ambient conditions (many locations now experience at least one such day per year), and our deployment-focused comparisons therefore target typical ambient ranges rather than event-driven extremes. Additionally, very high smoke concentrations fall in known nonlinear response regimes for PurpleAir sensors, which would require specialized treatment beyond this study’s scope [12,13].

PM_2.5_ concentrations were corrected using the U.S.-wide calibration equation developed by Barkjohn et al., 2021 [6]:

$${\mathrm{Corrected}\;\mathrm{PM}}_{2.5}=({\mathrm{Raw}\;\mathrm{PM}}_{2.5}\;(\mathrm{CF}=1))\times 0.52-(\mathrm{Relative}\;\mathrm{Humidity}\times 0.085)+5.71$$

To classify monitoring sites as urban or rural, we used the U.S. Census Bureau’s 2018 Urbanized Areas shapefile. This dataset delineates urbanized areas (population ≥ 50,000) and urban clusters (population 2500–49,999) at a national scale. We imported the shapefile into R and spatially joined it with sensor coordinates. Sensors located within an urbanized area or urban cluster polygon were categorized as “urban,” while those outside these boundaries were classified as “rural.”

## 3. Results

### 3.1. Analytic Sample

Publicly available data spanning 2016–2025 were initially downloaded for the U.S. The raw dataset contained n = 28,267 observations. After excluding sensors lacking location information, the dataset comprised n = 28,240 observations. Restricting the dataset to outdoor sensors reduced the total to n = 21,592. After removing sensors with missing geocoordinate information, the sample size was n = 17,511. Finally, removing observations for Puerto Rico and U.S. Virgin Islands yielded a final analytic dataset of n = 17,474 observations.

### 3.2. Geographic Distribution of PM_2.5_ Sensors

**Figure 1:**
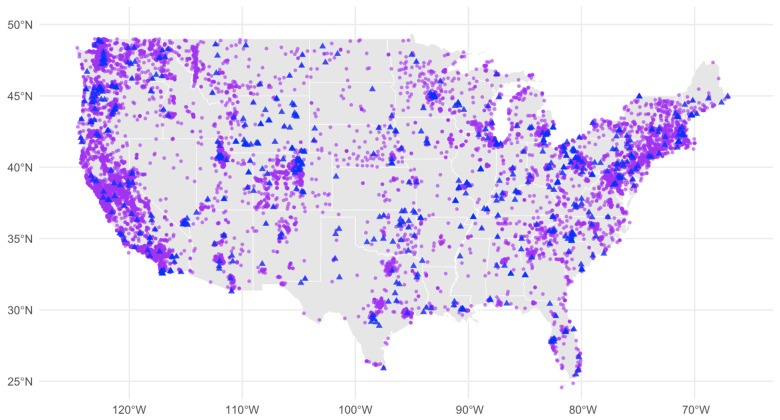
Geographic Distribution of PM_2.5_ Sensors: PurpleAir (purple points) and EPA FRM/FEM (blue triangles) Monitors Across the United States.

Figure 1 displays spatial distribution of publicly shared PurpleAir PM_2.5_ sensors (purple points) and U.S. EPA Federal Reference Method (FRM)/Federal Equivalent Method (FEM) PM_2.5_ monitors (blue triangles) across the United States. The figure highlights the dense, crowdsourced coverage of PurpleAir contrasted with the sparser regulatory-grade FRM/FEM network. This map provides context for our deployment-longevity analysis and the state-level illustrative comparisons presented later in the manuscript.

### 3.3. Deployment Longevity by Region and State

**Figure 2:**
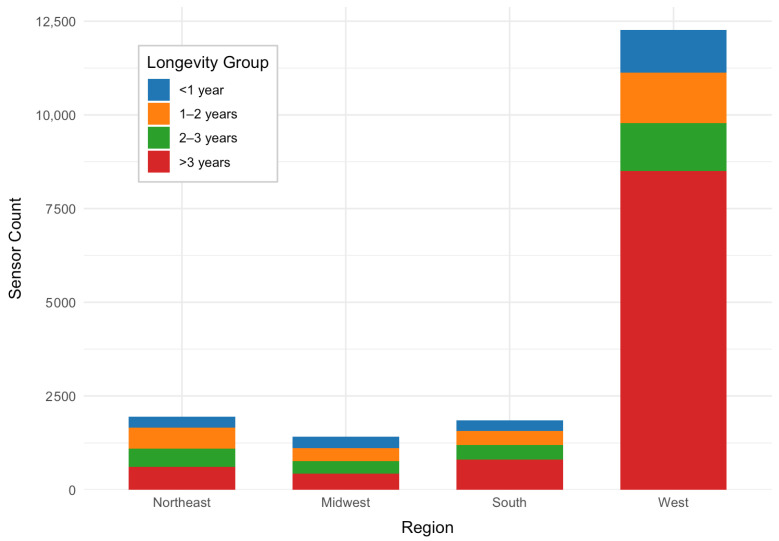
Sensor Deployment Longevity by Region, 2016–2025.

Sensor deployment longevity varied substantially across U.S. Census regions between 2016 and 2025 (Figure 2). The West region exhibited by far the highest number of PurpleAir sensors, exceeding 12,000 units, with the majority deployed for more than three years. In contrast, the Northeast and Midwest regions had substantially lower sensor counts—each with fewer than 2000 sensors overall—and comparatively more even distributions across longevity categories. The South showed slightly higher deployment than the Northeast and Midwest, with a noticeable share of sensors operating beyond three years but still far fewer than in the West. Across all regions, sensors deployed for more than three years represented the largest longevity category, while short-term deployments lasting less than one year were consistently the smallest group. These patterns indicate that long-term sensor retention is common nationwide, particularly in the West, potentially reflecting stronger adoption, sustained maintenance, and higher levels of community engagement with air quality monitoring in that region.

Table 2 summarizes sensor distribution across U.S. states by deployment duration. Nationwide, most sensors have been active for more than three years (n = 10,347; 59.2%), followed by deployments of 1–2 years (n = 2627; 15.0%), 2–3 years (n = 2491; 14.3%), and less than one year (n = 2009; 11.5%). California, Washington, and Oregon account for the largest sensor counts (n = 7138; 1604; and 1064, respectively), with a strong majority in each exceeding three years of operation. In contrast, states such as Mississippi (n = 10), Rhode Island (n = 29), and South Dakota (n = 23) have the fewest sensors, with deployments more evenly distributed across categories. Overall, sensor coverage and longevity vary widely, highlighting regions with extensive long-term monitoring alongside areas with limited adoption.

**Figure 3:**
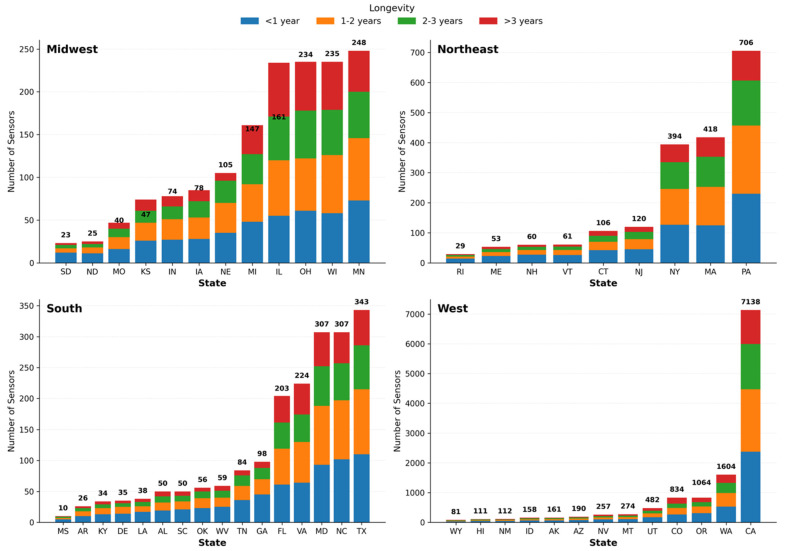
Sensor Deployment Longevity by Region and State, 2016–2025.

Sensor longevity patterns differ markedly by region (Figure 3). In the Northeast, Pennsylvania leads with over 700 sensors, most deployed for more than three years, while Massachusetts and New York also show high counts dominated by long-term installations. Vermont, New Hampshire, and Rhode Island have far fewer sensors and shorter deployments. In the Midwest, counts range from sparse networks in South Dakota and North Dakota to larger deployments in Illinois, Ohio, Michigan, and Minnesota, where long-term sensors predominate. The South exhibits moderate adoption with substantial variation: Texas and North Carolina have the highest counts, followed by Florida and Virginia, while many states maintain small networks with few long-duration sensors. The West shows the greatest disparity, driven by California’s more than 7000 sensors—most active for over three years—alongside sizable networks in Washington and Oregon and smaller deployments elsewhere. Across all regions, sensors deployed for more than three years consistently represent the largest category, indicating strong retention once installed.

### 3.4. Illustrative PM_2.5_ Summaries by Deployment Duration and Urban–Rural Classification in Four Exemplar States

Mean PM_2.5_ concentrations varied considerably by sensor deployment duration, though patterns differed across states (Table 3). California exhibited relatively consistent concentrations across all deployment durations (6.25–7.58 µg/m^3^), with sensors operating <1 year recording the highest mean (7.58 µg/m^3^) and maximum (23.7 µg/m^3^) values. Minnesota showed a different pattern, with sensors operating for 2–3 years exhibiting the highest mean concentration (8.01 µg/m^3^) compared to both shorter (<1 year: 6.99 µg/m^3^) and longer (>3 years: 7.61 µg/m^3^) deployment durations.

Pennsylvania indicataed a clear decreasing trend, with mean concentrations declining from 9.73 µg/m^3^ in sensors operating <1 year to 6.57 µg/m^3^ in those operating >3 years—a 32% reduction. Texas exhibited both the highest absolute PM_2.5_ levels and the most pronounced duration-related pattern among all states analyzed. Sensors operating <1 year recorded markedly elevated concentrations (mean: 12.7 µg/m^3^; range: 10.1–18.2 µg/m^3^), nearly double the mean observed in sensors operating >3 years (6.39 µg/m^3^). This substantial temporal variability likely reflects either deployment in response to episodic pollution events or capture of localized emission sources during the monitoring period.

Urban–rural differences in annual average PM_2.5_ concentrations revealed distinct state-specific patterns (Table 4). California and Minnesota showed relatively modest urban–rural differences in mean concentrations. In California, urban sensors recorded slightly higher means than rural sensors (6.91 vs. 6.25 µg/m^3^), with urban sites also exhibiting higher maximum values (16.80 vs. 14.90 µg/m^3^). Minnesota displayed similar mean concentrations between urban and rural locations (7.30 vs. 6.99 µg/m^3^), though urban sites recorded substantially higher maximum values (10.60 vs. 8.08 µg/m^3^).

In contrast, Pennsylvania and Texas exhibited elevated rural PM_2.5_ concentrations. Pennsylvania’s rural sensors reported a mean of 9.73 µg/m^3^ compared to 7.84 µg/m^3^ in urban areas, a 24% difference suggesting potential localized sources outside major metropolitan areas. Texas displayed the most pronounced urban–rural disparity among all states examined: rural sensors averaged 12.70 µg/m^3^—62% higher than the urban mean of 7.86 µg/m^3^—and reached a maximum of 18.20 µg/m^3^. The consistently elevated rural concentrations in Texas, combined with the narrow range observed in urban areas (4.28–10.20 µg/m^3^), suggest persistent rural pollution sources distinct from typical urban emission patterns.

We explored the feasibility of comparing PurpleAir sensor data with EPA monitoring data by identifying sensors that had collected data for at least three years with a completion rate above 75%, a threshold chosen to meet U.S. EPA data completeness standards—meaning the sensor recorded at least 75% of its expected hourly or daily measurements over the period—ensuring that the PurpleAir data could be reliably compared with reference monitors for regulatory purposes [14]. However, only a very small number of sensors met this criterion, limiting our ability to perform a meaningful long-term comparison. This scarcity likely reflects several factors: the voluntary nature of PurpleAir deployments, which often results in intermittent operation or sensor removal; lack of standardized maintenance protocols; and environmental conditions that can cause sensor downtime or data gaps [15,16]. Maintaining high data completeness over multi-year periods is inherently challenging for community-based sensor networks. Wallace et al. [17] noted that strict quality assurance measures substantially reduced usable data in their study. Unlike regulatory monitors, which operate under standardized QA/QC protocols, PurpleAir sensors are privately owned and may be designated as private, lack formal maintenance schedules, and are not subject to oversight, making sustained multi-year completeness difficult [17]. These limitations underscore the need for structured maintenance guidance and automated alerts to improve long-term data continuity.

As an explanatory analysis, we compared daily mean PM_2.5_ from one PurpleAir sensor with an EPA regulatory monitor located ≈100 m away near an airport and observed a positive but weak association (Pearson r = 0.231). The divergence likely reflects a combination of micro-environmental differences (the sites were near an airport with rapidly changing emissions and wind/turbulence), micro-siting/placement effects over tens of meters, and methodological differences between an optical, humidity-sensitive sensor (after correction) and a reference-grade instrument. Because this analysis is based on a single qualifying pair and does not stratify by wind sector or event conditions, it should be viewed as an illustrative demonstration of hyper-local variability.

## 4. Discussion

To guide interpretation and avoid overstating scope, we distinguish descriptive findings—network deployment patterns, spatial coverage, and sensor longevity—from hypothesis-generating implications for exposure assessment or policy. Descriptive results indicate where and how long sensors operate; they are not causal or regulatory claims. Any policy-relevant implication would require additional validation (collocation, completeness thresholds, event stratification, and standardized calibration workflows).

### 4.1. Key Insights

Our analysis answers a core question: Do publicly shared PurpleAir sensors remain deployed and discoverable long enough—and where—to support multi-year use cases? Nationally, we find a large, persistent community network: many devices remain online for more than three years, suggesting that multi-year applications are feasible in substantial parts of the country. At the same time, coverage is uneven. The West—especially California, Washington, and Oregon—dominates long-duration deployments, whereas many states in the Northeast, Midwest, and South have sparse networks. These patterns underscore persistent gaps in low-cost sensor availability, which may limit air quality monitoring in rural or underserved areas and constrain the representativeness of exposure data that can be used for epidemiological research [11,18]. Duration-stratified summaries in four exemplar states (CA, MN, PA, TX) also show that short-duration sensors often capture higher means or extremes, consistent with event-driven or opportunistic installations; thus, interpreting concentration summaries likely would require attention to deployment timing and context. Urban–rural contrasts are state-specific: in some states, rural areas exhibit elevated PM2.5 relative to urban areas, suggesting the value of non-metro coverage.

Elevated rural PM_2.5_ levels in Texas and Pennsylvania underscore the need for expanded sensor coverage beyond metropolitan areas. However, these patterns should be interpreted cautiously: the present analysis does not disentangle true environmental signals from deployment bias (e.g., local event-driven or seasonal placements) or sensor-related artifacts (including correction performance under smoke/high humidity). Accordingly, we do not attempt source attribution here; careful interpretation that acknowledges deployment context, siting, and methodological nuances is essential before drawing strong conclusions based on our exploratory analyses.

What this study does is establish a national baseline of where publicly shared sensors exist and how long they persist on the public network, thereby identifying regions and states that already support multi-year analyses and those where persistence or coverage remains a limiting factor. What it does not do is assert regulatory comparability, causal attribution, or comprehensive exposure modeling. Those aims need collocated reference data, completeness thresholds, event stratification, and standardized calibration workflows that are beyond the remit of a deployment-focused study.

An exploratory comparison between a single PurpleAir sensor and a nearby EPA monitor (~100 m) yielded a positive but weak association (r = 0.231). This example illustrates a central trade-off: hyper-local sensors can detect fine-scale variability that a single regulatory monitor cannot resolve, yet that same granularity complicates one-to-one alignment without careful siting, wind-sector/event stratification, and method harmonization. The example should therefore be viewed as a demonstration of hyper-local variability, not as evidence for or against regulatory comparability.

### 4.2. Limitations

Several limitations should be acknowledged. First, our deployment metric (first-seen to last-seen) captures network tenure, not verified continuous operation; intermittent downtime can overstate availability. Second, even with widely used corrections, extreme smoke or dust conditions can introduce nonlinear response, so our QC emphasizes baseline ranges rather than extremes. Measurement biases related to environmental conditions may persist despite applying correction algorithms, as these models often fail to account for extreme pollution events or unique atmospheric conditions. For example, the EPA correction improves agreement with regulatory monitors for urban and smoke aerosols but underestimates PM2.5 during dust events and at very high concentrations (>600 µg/m^3^) [15]. Recent studies propose localized or event-specific calibration approaches to address these gaps [17,19], yet a systematic framework for applying such methods across diverse regions remains lacking. This underscores the need for geographically tailored correction models to ensure reliable data for health research and policy applications. Third, the four state exemplars are high-coverage cases used for illustration, not region-wide generalizations.

### 4.3. Future Opportunities

Although developing a nationwide framework to integrate sensor data into exposure models remains challenging, emerging evidence shows that low-cost networks such as PurpleAir can enhance epidemiological exposure assessment when properly calibrated and incorporated into spatiotemporal models. Bi et al. [20] used publicly available PurpleAir PM2.5 measurements alongside regulatory “gold-standard” monitors to improve exposure predictions for participants in the Adult Changes in Thought–Air Pollution (ACT-AP) cohort. Incorporating calibrated PurpleAir data into exposure models increased external validation performance (higher R^2^ and lower RMSE) and revealed sharper spatial gradients, enhancing representation of fine-scale variability in PM2.5 relevant to health studies. The authors also proposed metrics to assess the representativeness of sensor locations relative to cohort residences, underscoring the importance of thoughtful deployment to avoid spatial bias [20]. Similarly, Coker et al. [21] employed a PurpleAir sensor in Rio Branco, Brazil, to examine associations between PM2.5 and daily respiratory hospitalizations. Corrected sensor data produced stronger, more accurate effect estimates than uncorrected readings, demonstrating that calibrated low-cost sensors can support epidemiological analyses in regions lacking regulatory monitors [21]. Collectively, these findings highlight the potential of PurpleAir data to enhance high-resolution exposure models, provided robust calibration and validation strategies are applied.

Here are some next steps for consideration. First, prioritize targeted augmentation and collocation in low-coverage states to improve both spatial equity and calibration. Second, adopt standardized platform-side curation (plausibility checks, completeness/persistence metrics, and event tagging) with published confidence labels to facilitate downstream use. Third, machine learning can be used to further utilize the low-cost sensor data. For example, Lu and colleagues developed a high-resolution (500 × 500 m) model of hourly PM2.5 concentrations in Los Angeles County by integrating quality-controlled PurpleAir low-cost sensor data with spatial predictors in a machine-learning framework. The model showed strong predictive performance (cross-validated R^2^ up to 0.93) and captured fine-scale spatial and temporal variability, including wildfire episodes, demonstrating the potential value of calibrated low-cost sensors for exposure assessment in environmental health studies [22]. Additionally, emerging ML techniques offer promise for improving calibration by capturing nonlinear relationships between environmental variables and pollutant concentrations, but these approaches remain non-standardized and their transferability across regions is uncertain [23].

Finally, clarifying roles helps improve utility. As citizen-science use grows, guidance for non-experts (e.g., outdoor placement, power/connectivity, weather protection) is helpful, but non-professional users would not be the primary stewards of research- or policy-relevant deployment. Their reasonable expectation may be to use convenient, accessible devices; accordingly, producing decision-useful data should rest chiefly with device developers, data platforms, and sponsoring programs. In practice, low-cost sensors deliver the most value as a supplement to the regulatory network when curated by researchers or agencies under pre-specified QA/QC—automated plausibility checks, minimum completeness/persistence thresholds, event tagging (e.g., smoke/dust), vetted corrections, and clear uncertainty labels. This system-focused approach leverages high spatial density for hyper-local screening, siting reconnaissance, and short-duration decision support, while FRM/FEM monitors remain the foundation for compliance and long-term trend assessment—thereby improving utility and reliability without shifting obligations onto casual users.

In conclusion, publicly shared PurpleAir sensors are numerous and often persistent, yet coverage remains uneven, creating predictable gaps in representativeness. By answering where and how long community sensors operate, this study provides the baseline evidence needed to plan multi-year analyses and targeted expansions. When paired with standardized QA/QC and collocation, curated low-cost data can supplement regulatory networks by adding hyper-local detail, while FRM/FEM monitors continue to anchor regulatory compliance and long-term trend assessment.

## Figures and Tables

**Table 1:** PurpleAir Sensor Field Descriptions.

Field Name	Description
date_created	The date and time when the sensor data entry was initially recorded.
last_seen	The most recent timestamp indicating when the sensor was active or online.
location_type	Type of sensor location (e.g., indoor, outdoor).
humidity	Relative humidity (%) measured at the sensor location.
temperature	Ambient temperature (°C or °F) at the sensor location.
pressure	Atmospheric pressure (hPa or inHg) at the sensor location.
pm2.5_cf_1	Particulate matter concentration (µg/m^3^) for particles ≤ 2.5 µm, corrected using the CF = 1 algorithm (using a basic Conversion Factor = 1; uncorrected for humidity or calibration).

**Table 2:** Publicly Available Sensor Counts (%) by State and Deployment Longevity, 2016–2025.

State	<1 Year	1–2 Years	2–3 Years	>3 Years	Total
Alabama (AL)	4 (8.0)	7 (14.0)	10 (20.0)	29 (58.0)	50
Alaska (AK)	31 (19.3)	20 (12.4)	22 (13.7)	88 (54.7)	161
Arizona (AZ)	40 (21.1)	48 (25.3)	27 (14.2)	75 (39.5)	190
Arkansas (AR)	6 (23.1)	10 (38.5)	1 (3.8)	9 (34.6)	26
California (CA)	596 (8.3)	610 (8.5)	462 (6.5)	5470 (76.6)	7138
Colorado (CO)	77 (12.1)	90 (14.2)	85 (13.4)	382 (60.3)	634
Connecticut (CT)	9 (8.5)	7 (6.6)	32 (30.2)	58 (54.7)	106
Delaware (DE)	3 (8.6)	10 (28.6)	5 (14.3)	17 (48.6)	35
District of Columbia (DC)	7 (11.9)	24 (40.7)	15 (25.4)	13 (22.0)	59
Florida (FL)	26 (19.0)	45 (32.8)	28 (20.4)	38 (27.7)	137
Georgia (GA)	16 (16.3)	14 (14.3)	21 (21.4)	47 (48.0)	98
Hawaii (HI)	7 (6.3)	24 (21.6)	40 (36.0)	40 (36.0)	111
Idaho (ID)	18 (11.4)	41 (25.9)	16 (10.1)	83 (52.5)	158
Illinois (IL)	25 (15.5)	41 (25.5)	44 (27.3)	51 (31.7)	161
Indiana (IN)	22 (29.7)	13 (17.6)	12 (16.2)	27 (36.5)	74
Iowa (IA)	10 (12.8)	14 (17.9)	11 (14.1)	43 (55.1)	78
Kansas (KS)	12 (25.5)	8 (17.0)	10 (21.3)	17 (36.2)	47
Kentucky (KY)	4 (11.8)	7 (20.6)	14 (41.2)	9 (26.5)	34
Louisiana (LA)	8 (21.1)	2 (5.3)	8 (21.1)	20 (52.6)	38
Maine (ME)	16 (30.2)	9 (17.0)	13 (24.5)	15 (28.3)	53
Maryland (MD)	10 (4.5)	25 (11.2)	42 (18.8)	147 (65.6)	224
Massachusetts (MA)	33 (7.9)	178 (42.6)	70 (16.7)	137 (32.8)	418
Michigan (MI)	49 (20.9)	48 (20.4)	80 (34.0)	58 (24.7)	235
Minnesota (MN)	64 (25.8)	63 (25.4)	39 (15.7)	82 (33.1)	248
Mississippi (MS)	4 (40.0)	2 (20.0)	3 (30.0)	1 (10.0)	10
Missouri (MO)	2 (5.0)	8 (20.0)	8 (20.0)	22 (55.0)	40
Montana (MT)	39 (14.2)	62 (22.6)	50 (18.2)	123 (44.9)	274
Nebraska (NE)	15 (14.3)	40 (38.1)	37 (35.2)	13 (12.4)	105
Nevada (NV)	18 (7.0)	26 (10.1)	17 (6.6)	196 (76.3)	257
New Hampshire (NH)	11 (18.3)	8 (13.3)	9 (15.0)	32 (53.3)	60
New Jersey (NJ)	20 (16.7)	12 (10.0)	48 (40.0)	40 (33.3)	120
New Mexico (NM)	18 (16.1)	18 (16.1)	11 (9.8)	65 (58.0)	112
New York (NY)	61 (15.5)	89 (22.6)	139 (35.3)	105 (26.6)	394
North Carolina (NC)	19 (6.2)	70 (22.8)	76 (24.8)	142 (46.3)	307
North Dakota (ND)	3 (12.0)	7 (28.0)	8 (32.0)	7 (28.0)	25
Ohio (OH)	52 (22.2)	70 (29.9)	52 (22.2)	60 (25.6)	234
Oklahoma (OK)	10 (17.9)	18 (32.1)	18 (32.1)	10 (17.9)	56
Oregon (OR)	99 (9.3)	179 (16.8)	177 (16.6)	609 (57.2)	1064
Pennsylvania (PA)	122 (17.3)	234 (33.1)	155 (22.0)	195 (27.6)	706
Rhode Island (RI)	10 (34.5)	2 (6.9)	9 (31.0)	8 (27.6)	29
South Carolina (SC)	17 (34.0)	7 (14.0)	8 (16.0)	18 (36.0)	50
South Dakota (SD)	0 (0.0)	5 (21.7)	12 (52.2)	6 (26.1)	23
Tennessee (TN)	20 (22.2)	11 (12.2)	22 (24.4)	37 (41.1)	90
Texas (TX)	70 (20.4)	69 (20.1)	32 (9.3)	172 (50.1)	343
Utah (UT)	32 (6.6)	50 (10.4)	55 (11.4)	345 (71.6)	482
Vermont (VT)	11 (18.0)	15 (24.6)	11 (18.0)	24 (39.3)	61
Virginia (VA)	29 (14.3)	22 (10.8)	65 (32.0)	87 (42.9)	203
Washington (WA)	156 (9.7)	162 (10.1)	300 (18.7)	986 (61.5)	1604
West Virginia (WV)	26 (31.0)	29 (34.5)	17 (20.2)	12 (14.3)	84
Wisconsin (WI)	44 (29.9)	31 (21.1)	32 (21.8)	40 (27.2)	147
Wyoming (WY)	8 (9.9)	23 (28.4)	13 (16.0)	37 (45.7)	81
Total	2009 (11.5)	2627 (15.0)	2491 (14.3)	10,347 (59.2)	17,474

**Table 3:** PM_2.5_ Concentration (µg/m^3^) for California, Pennsylvania, Minnesota, and Texas by Sensor Deployment Longevity.

State (Affiliated Region)	Duration	Minimum	Mean	Maximum
California (West)	<1 year	1.93	7.58	23.70
	1–2 years	1.42	7.04	16.80
	2–3 years	2.69	6.25	14.90
	>3 years	0.78	6.62	16.70
Minnesota (Midwest)	<1 year	6.09	6.99	8.08
	1–2 years	3.39	7.30	10.60
	2–3 years	4.91	8.01	10.10
	>3 years	5.77	7.61	9.20
Pennsylvania (Northeast)	<1 year	3.27	9.73	11.90
	1–2 years	4.32	7.84	13.20
	2–3 years	3.77	7.39	12.10
	>3 years	2.09	6.57	9.06
Texas (South)	<1 year	10.10	12.70	18.20
	1–2 years	4.28	7.86	10.20
	2–3 years	5.36	7.21	8.87
	>3 years	5.80	6.39	7.12

**Table 4:** PM_2.5_ Concentration (µg/m^3^) for California, Pennsylvania, Minnesota, and Texas by Urban vs. Rural Status.

State	Location	Minimum	Mean	Maximum
California (West)	Urban	1.42	6.91	16.80
	Rural	2.69	6.25	14.90
Minnesota (Midwest)	Urban	3.39	7.30	10.60
	Rural	6.09	6.99	8.08
Pennsylvania (Northeast)	Urban	4.32	7.84	13.20
	Rural	3.27	9.73	11.90
Texas (South)	Urban	4.28	7.86	10.20
	Rural	10.10	12.70	18.20

## Data Availability

The original contributions presented in this study are included in Section 2. Further inquiries can be directed to the corresponding author.
